# Accelerated evolution of 3'avian FOXE1 genes, and thyroid and feather specific expression of chicken FoxE1

**DOI:** 10.1186/1471-2148-11-302

**Published:** 2011-10-15

**Authors:** Sergey Yu Yaklichkin, Diana K Darnell, Maricela V Pier, Parker B Antin, Sridhar Hannenhalli

**Affiliations:** 1 Penn Center for Bioinformatics, 1424 Blockley Hall, 423 Guardian Drive, University of Pennsylvania, Philadelphia, PA 19104 USA; 2 Department of Cellular and Molecular Medicine, University of Arizona, PO Box 245217, 1656 E. Mabel, Tucson, AZ 85724 USA; 3 Center for Bioinformatics and Computational Biology, University of Maryland, College Park, 3104G Biomolecular Sciences Building (#296), University of Maryland, College Park, MD 20742 USA

## Abstract

**Background:**

The forkhead transcription factor gene E1 (FOXE1) plays an important role in regulation of thyroid development, palate formation and hair morphogenesis in mammals. However, avian *FOXE1 *genes have not been characterized and as such, codon evolution of FOXE1 orthologs in a broader evolutionary context of mammals and birds is not known.

**Results:**

In this study we identified the avian *FOXE1 *gene in chicken, turkey and zebra finch, all of which consist of a single exon. Chicken and zebra finch *FOXE1 *are uniquely located on the sex-determining Z chromosome. In situ hybridization shows that chicken *FOXE1 *is specifically expressed in the developing thyroid. Its expression is initiated at the placode stage and is maintained during the stages of vesicle formation and follicle primordia. Based on this expression pattern, we propose that avian *FOXE1 *may be involved in regulating the evagination and morphogenesis of thyroid. Chicken *FOXE1 *is also expressed in growing feathers. Sequence analysis identified two microdeletions in the avian *FOXE1 *genes, corresponding to the loss of a transferable repression domain and an engrailed homology motif 1 (Eh1) C-terminal to the forkhead domain. The avian *FOXE1 *proteins exhibit a significant sequence divergence of the C-terminus compared to those of amphibian and mammalian *FOXE1*. The codon evolution analysis (dN/dS) of *FOXE1 *shows a significantly increased dN/dS ratio in the avian lineages, consistent with either a relaxed purifying selection or positive selection on a few residues in avian FOXE1 evolution. Further site specific analysis indicates that while relaxed purifying selection is likely to be a predominant cause of accelerated evolution at the 3'-region of avian FOXE1, a few residues might have evolved under positive selection.

**Conclusions:**

We have identified three avian *FOXE1 *genes based on synteny and sequence similarity as well as characterized the expression pattern of the chicken *FOXE1 *gene during development. Our evolutionary analyses suggest that while a relaxed purifying selection is likely to be the dominant force driving accelerated evolution of avian *FOXE1 *genes, a few residues may have evolved adaptively. This study provides a basis for future genetic and comparative biochemical studies of FOXE1.

## Background

*FOXE1 *is a member of the large and evolutionarily ancient family of forkhead domain-containing transcriptional regulators, which are involved in a variety of developmental and physiological processes in organisms from yeast to mammals [1]. *FOXE1*, previously termed thyroid transcription factor-2, (TTF-2) was originally isolated by screening a rat cDNA library [2]. The FOXE1 protein was shown to bind specifically to the thyroglobulin promoter and function as a transcriptional repressor [2,3]. During mouse embryogenesis FoxE1 is expressed in developing thyroid, Rathke's pouch, palate, tongue, epiglottis, pharynx, and oesophagus and in the epithelium of the pharyngeal wall and arches [2,4]. FOXE1 transcripts are also found in the hair follicle and are regulated by sonic hedgehog signaling in the human and mouse [5,6]. Consistent with its expression pattern, *FOXE1*-null mutant mice exhibit either a sublingual or completely absent thyroid gland, cleft palate and abnormal hair structure and growth [7,6]. Similarly, mutations in the forkhead DNA-binding domain of the human *FOXE1 *gene cause thyroid agenesis, cleft palate and choanal atresia similar to the phenotype observed in *FOXE1*-null mutant mice [8]. Taken together, the crucial role of *FOXE1 *in thyroid formation, palate, and hair development is well established in placental mammals.

Expression of *FOXE1 *orthologs in other vertebrates is similar to their mammalian counterparts. For example, in the *Xenopus *embryo, foxe1 is expressed in the developing thyroid, pituitary mesoderm of brachial arches and the pharyngeal endoderm [9]. In the zebrafish embryo foxe1 is expressed in the thyroid, pharynx, and pharyngeal skeleton [10]. In addition, the gene is strongly expressed in the gill and weakly expressed in the brain, eye, and heart in adult zebrafish. However, in contrast to the role of *FOXE1 *in placental mammals, a loss-of-function study demonstrated that zebrafish foxe1 is not required for the thyroid formation but is necessary for chondrogenesis during pharyngeal skeleton formation [10]. These data suggest that FOXE1 may have acquired the role in the regulation of thyroid development during the evolution of tetrapods, or may have lost this role in the fish lineage. On the other hand, *FOXE1 *is involved in the regulation of hair morphogenesis, which is a relatively recent skin organ, appearing in the mammalian lineage [6]. This suggests that *FOXE1 *has acquired a novel regulatory function in the mammalian lineage. Taken together, the data supports substantial functional evolution of FOXE1 during vertebrate evolution.

Despite progress in understanding the function of the mammalian, amphibian and fish *FOXE1 *genes, nothing is known about *FOXE1 *gene in birds. The study of *FOXE1 *of birds can help fill the missing link and provide important insights into the evolution of this gene in vertebrates. Here, we have identified *FOXE1 *genes in multiple avian species and characterized its expression pattern during chicken development using *in situ *hybridization. Our data shows that chicken *FOXE1 *expression is limited to developing thyroid and feathers. We also observe a significant sequence divergence of the N- and C- terminus of the avian FOXE1 proteins and a loss of two repressive domains. Our codon analysis (dN/dS) of avian *FOXE1 *genes suggests that relaxed purifying selection, or alternatively, positive selection in a subset of residues, might have driven sequence divergence of the avian FOXE1 C-terminus.

## Results

### Identification and characterization of FoxE1 genes of chicken, zebra finch, and turkey

**Figure 1:**
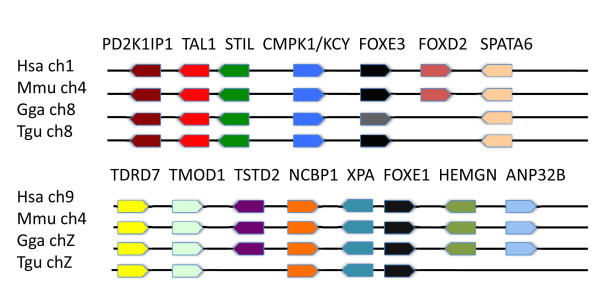
**Syntenic alignments of putative avian FOXE3 (Chromosome 8) and the FOXE1 homolog (Chromosome Z) are identified with human (*Hsa*), mouse (*Mmu*), chicken (*Gga*), and zebra finch (*Tgu*) FOXE3 and FOXE1, respectively**.

Chicken *FOXE1 *is currently listed in the ensembl database as ENSGALG00000023293 located at Chromosome 8: 22,697,482-22,698,342. Although *FOXE1 *genes in other species are listed as orthologs for this gene, the lack of synteny with other FoxE1 genes indicates that this region is not homologous to FOXE1 but with FoxE3 (Figure 1). A true homolog (with appropriate synteny) of FOXE1 has not been previously reported in the chicken. We searched for the *FOXE1 *gene in the chicken genome database using a mouse *FOXE1 *gene as the query sequence in the BLAST search. A genomic region of the highest homology to the query sequence was found on the chicken BAC clone CH261-25P17, representing a part of the sex-determining Z chromosome. The expression of the chicken *FOXE1 *gene was confirmed with RT-PCR using a chicken embryonic cDNA library (data not shown). Analysis of the region of homology revealed an uninterrupted ORF of 873 bps, encoding a 290 aa protein sequence with a molecular weight of 30617 Da (Additional File 1, Figure S1). The deduced protein sequence shows the highest similarity to the FOXE1 forkhead domain (53-151aa) with ~88% identity, whereas the C-terminus outside the forkhead domain shows a much reduced similarity (less than 30%). The C-terminus of the chicken FOXE1 protein is shorter (139 aa) than those of orthologous FOXE1 proteins. Moreover, the N-terminal 41 aa of chicken FOXE1 has no significant similarity to the N-terminal domains of other FOXE1 proteins. The chicken *FOXE1 *gene is extremely GC-rich (overall GC-content = 78%), and the highest GC-content is in the 3'-region of the gene, encoding the C-terminus (GC-content = 85%) (Additional File 1, Figure S2). The syntenic arrangement of surrounding genes confirmed that this was *FOXE1 *(Figure 1). Thus, we identified the chicken *FOXE1 *gene consisting of a single exon similar to orthologous genes.

We further searched for the presence of a zebra finch *FOXE1 *using a chicken *FOXE1 *gene as the query against the genomic dataset of zebra finch. The blast search identified a genomic region of 588 bps on the minus strand of Z chromosome with the coordinates 31552116-31551529 bps, (genome version WUGSC 3.2.4/taeGut1) exhibiting 88% similarity to the chicken *FOXE1 *nucleotide sequence. An ORF was identified in this chromosomal region (Additional File 1, Figure S3), and the corresponding protein sequence exhibited the highest homology to FOXE1 proteins. Thus, this similarity clearly indicated that the indentified region is a zebra finch ortholog of chicken FOXE1 (Additional File 1, Figure S4). However, the zebra finch genomic sequence lacked a 5' - part, encoding an entire N-terminus and a 5' - portion of the forkhead domain because of a gap in the sequence of the Z chromosome. Nevertheless, the region represents a major portion of the gene, encoding a portion of the forkhead domain and an entire C-terminus.

To better characterize *FOXE1 *in avian lineages, we further searched for *FOXE1 *gene in the reference genome of turkey. However, despite the recent sequencing of the turkey genome, the *FOXE1 *gene sequence was not identified. Upon closer inspection, we concluded that the corresponding genomic region is missing from the turkey reference genome. Therefore, we identified the *FOXE1 *gene from turkey by direct genomic PCR amplification and sequencing. Remarkably, the putative turkey *FOXE1 *gene exhibited 97% identity to the chicken gene, which is consistent with the reported high similarity of chicken and turkey genomes [11].

Interestingly, avian *FOXE1 *genes in both chicken and zebra finch are located on the sex-determining Z chromosome, which is distinct from the chromosomal location of vertebrate orthologs. Fish and mammalian *FOXE1 *are located on autosomal chromosomes (data not shown). This difference in localization indicates two possibilities: the avian *FOXE1 *was either part of the ancestral autosomal chromosome which has evolved into the Z chromosome in an ancestral amniote [12], or *FOXE1 *genomic locus was translocated onto the Z chromosome. Synteny between the chicken chromosome Z and human Chromosome 9, which includes the sex determining DMRT1, indicates that the chromosome Z evolved from the autosomal chromosome [13]. Human *FOXE1 *is located on chromosome 9. Therefore, a distinctive chromosomal localization of avian *FOXE1 *in birds is likely associated with the ancestral autosomal chromosome that subsequently evolved into the Z chromosome.

In summary, by synteny-based analysis of orthology in chicken, and by direct sequencing in turkey, we have identified the avian orthologs of mammalian *FOXE1 *gene, at least two of which are localized on the sex-determining Z chromosome.

### Expression of chicken FoxE1 gene is restricted to the developing thyroid and feathers

**Figure 2:**
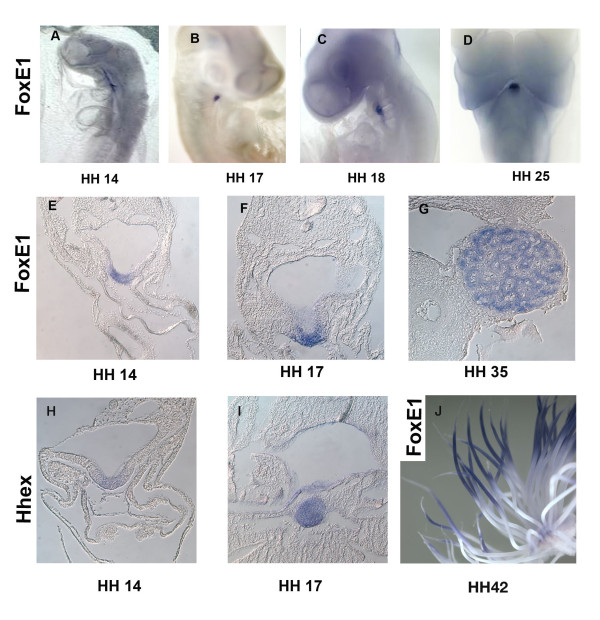
***FOXE1 *expression in developing chicken embryos**. Antero-lateral views showing whole mount *in situ *hybridization localization of *FOXE1 *transcripts in chicken embryos at stages 14 (A), 17 (B), and 18 (C); anterior view of the embryonic pharyngeal arches at stage 25 (D). Transverse sections showing *FOXE1 *expression at stages 14 (E), 17 (F), and 35 (G). *In situ *hybridization showing the localization of the thyroid expressed gene *HHEX *stages 14 (H) and 35 (I). *FOXE1 *is expressed in distal growing feathers by HH stage 42 (J).

To determine the expression pattern of *FOXE1 *during chicken embryogenesis, whole mount *in situ *hybridization was performed on chicken embryos spanning embryonic stages 3-42 [14] with a 504 bps antisense probe for *FOXE1*. Expression of *FOXE1 *was observed at but not before stage 14, and was restricted to the primordial thyroid placode (Figure 2A). At this stage the thyroid placode is forming by anterior bending of the pharynx floor, and is characterized by a low proliferative index [15]. *FOXE1 *expression is clearly visible when the placode is discernible (Figure 2E). At stage 17, the thyroid primordium is in an advanced stage of evagination, and expression of *FOXE1 *was more pronounced in the indentations rather than in the shoulders of the evagination (Figure 2F). At stage 18, expression of *FOXE1 *was restricted to the anterior pharynx at the level of the second pharyngeal arch (Figure 2C). By stage 19, the thyroid develops into a vesicle [14]. *FOXE1 *expression is maintained in the forming vesicle during budding off of the gland from the pharynx. *FOXE1 *expression is maintained at stage 25 (Figure 2D) when the thyroid separates from the pharynx and exhibits a dramatic change in shape that marks the beginning of bilobation [15]. At stage 35, the thyroid is invaded by vascular and connective tissues and strong expression of *FOXE1 *was observed throughout the follicle primordium (Figure 2G). This suggests that *FOXE1 *may be involved in the maturation of the gland as well. We confirmed this tissue as the developing thyroid using the transcription factor gene HHEX, which is expressed in the developing thyroid ([16] Figure 2H, I). *HHEX*, is important for proliferation of thyroid cell precursors and for thyroid morphogenesis [17]. *In situ *hybridization showed that expression domains of *FOXE1 *and *HHEX *are similar, clearly marking the developing thyroid. Although no expression of *FOXE1 *was detected in feather buds at stages 30-35, *FOXE1 *is expressed in distal growing feathers by HH stage 42 (Figure 2J). Interestingly, no chicken *FOXE1 *expression was observed in the developing pituitary or, palate, as has been reported for amphibian and murine *FOXE1 *genes, respectively. In conclusion, *in situ *hybridization analysis shows that chicken *FOXE1 *is a thyroid and feather-specific transcription factor, and suggests a possible role for *FOXE1 *in the evagination, and morphogenesis of the thyroid and feathers.

### Evolution of the avian FoxE1 proteins: a loss of a repressive domain and the Eh1 motif

**Figure 3:**
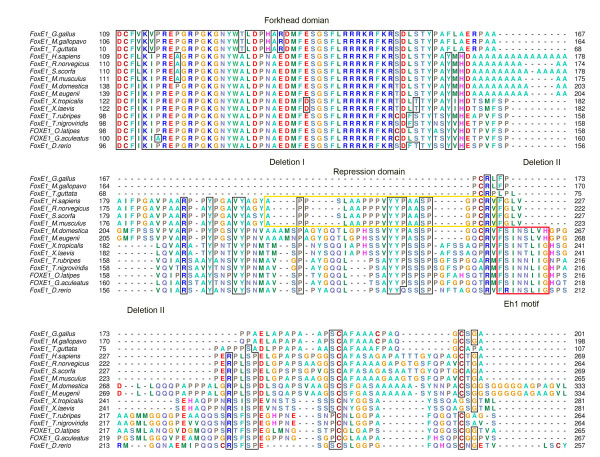
**Multiple sequence alignments showing portions of the forkhead domain and C-terminus of FOXE1 proteins**. (A) The avian FOXE1 proteins has lost the engrailed homology motif 1 (Eh1) and the aromatic repression domain during evolution, due to the two microdeletions, probably occurring in the lineage of birds. The aromatic repression domain is highlighted with the yellow box and the Eh1 motif with the red box in the aligned protein sequences. The numbers on the left indicate the positions of amino acid residues in the respective sequences. The positions for FOXE1 of *T. guttata *(zebra finch) are enumerated from the forkhead domain due to the missing N-terminal sequences.

To investigate the basis for the size reduction and sequence divergence of the avian FOXE1 proteins relative to mammalian and amphibian counterparts, we constructed a multiple sequence alignment of all FOXE1 genes. The sequence alignment revealed two deletions at the C-terminus: a first deletion corresponding to 66 aa and a second deletion corresponding to 28 aa protein region (Figure 3). The first deletion resulted in an avian-specific loss of a 21 aa aromatic domain (Figure 3), which previously was shown to exhibit transcriptional repression activity in cell culture [3]. The second deletion led to the loss of sequences encoding almost the entire Eh1 motif and adjacent sequences except for a conserved phenylalanine residue, which is preserved in avian proteins. The Eh1 motif is a conserved eight amino acid sequence FSIN[TSN]L[IV][GH], which is present in a majority of foxe1 proteins of fish, amphibians and non-placental mammals [18]. Similarly, the loss of the Eh1 motif is observed in FOXE1 of placental mammals (Figure 3) (Yaklichkin, Kessler, unpublished data). The Eh1 motif is known to mediate physical interactions of FOX proteins with the Groucho/TLE co-repressors [19,20].

Additionally, a novel feature in the avian FOXE1 proteins is the presence of an N-terminal polyalanine repeat (Additional File 2, Figure S5), varying from five to nine alanine residues. The polyalanine repeat is also found in FOXE1 of mammals where it is distal to the forkhead domain, but is absent in FOXE1 proteins of amphibians and fish. This suggests that the avian polyalanine repeat arose independently in the avian lineage. The C-terminus of the chicken FOXE1 protein is also enriched with alanine, glycine, and proline residues (Additional File 1, Table S1), as well as short tandem repeats of proline and alanine (Additional File 1, Table S2; [21]). Proline and alanine-enriched domains are commonly found in transcriptional repressors [22]. This suggests that the avian FOXE1 protein may function as a transcriptional repressor. Thus, while there appears to be an avian-specific loss of two repressive domains in FOXE1 as a result of deletions, we also found an avian specific gain in proline-alanine repeats, which can potentially confer transcriptional repressive activity to avian FOXE1.

### Analysis of codon evolution in the avian lineage of *FOXE1*

**Figure 4:**
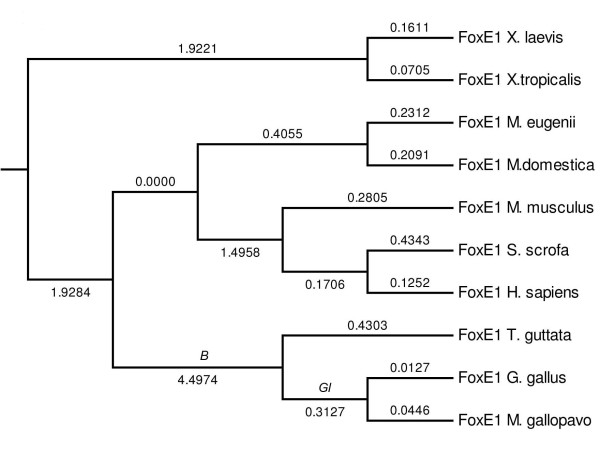
**Tree topology and the models used for the likelihood-ratio tests**. The numbers on the tree branches represent the mean number of substitutions per codon along the branch as estimated by the program Mega4. Branches *B (*common ancestor of birds) and *Gl *(common ancestor of *Galliformes*) were the lineages of the analysis in free branch models.

Next we investigated whether, consistent with a functional divergence, *FOXE1 *has experienced accelerated evolution (relaxed purifying selection, or positive selection) specifically in bird ancestry and specific bird lineages using *codeml *[23]. Multiple sequence alignment was constructed using three bird and seven other tetrapod FOXE1 sequences encoding a portion of the forkhead domain and an entire C-terminus, where most of the sequence divergence in the available sequence is concentrated. We first compared two models of evolution using a likelihood ratio test (LRT) [24]. In Model-0 (one-ratio model) all lineages were assumed to have the same evolutionary rate ω0 (dN/dS) in the gene phylogeny (Figure 4); the most likely estimate of ω0 in this case was 0.052. According to Model-1 (two-ratio model), the bird lineages and their ancestral lineage (a total of 5 branches) evolved at a rate of ω1 while all other lineages evolved at rate ω0. The estimates of ω0 and ω1 were 0.0386 and 0.1042, respectively, corresponding to a 2.7-fold increase in the evolutionary rate in the bird lineage. Model-1 had a significantly better fit to the data than Model-0 (*P *= 0.0009). In Model-2 (three-ratio model) we assumed that the common ancestor of *Galliformes *(the order of turkey and chicken), and turkey and chicken were evolving at rate ω2 and the common ancestor of the avian species and zebra finch were evolving at ω1, while all other lineages evolved with rate ω0. Comparing this model with the first model yielded a *P*-value = 0.003, with ω0 = 0.038, ω1 = 0.12, and ω2 = 0.083. In Model-3 (three-ratio model), we assumed ω2 for the zebra finch branch and ω1 for the four branches - common ancestor of all birds and the three *Galliformes *branches. Comparing this model with the first model resulted in a *P*-value = 0.0002, with ω0 = 0.038, ω1 = 0.12, and ω2 = 0.4801 corresponding to a 12.4-fold increase in the evolutionary rate in the zebra finch lineage. These results indicate that there is overall evidence of accelerated evolution in avian *FOXE1*. Thus, we conclude that *FOXE1 *is likely to have evolved either under relaxed purifying selection or restricted positive selection in a few specific residues in the avian lineage. Placental *FOXE1 *genes, which also lost the Eh1 motif, were tested for evolutionary rate by assigning ω1 to the placental branch, which resulted in ω0 = 0.0561 and ω1 = 0.0476. Thus, no increase of the evolutionary rate in *FOXE1 *of placental mammals is observed in our dataset. This suggests that overall, avian *FOXE1 *has experienced either relaxed purifying selection or positive selection on a few specific residues.

**Figure 5:**
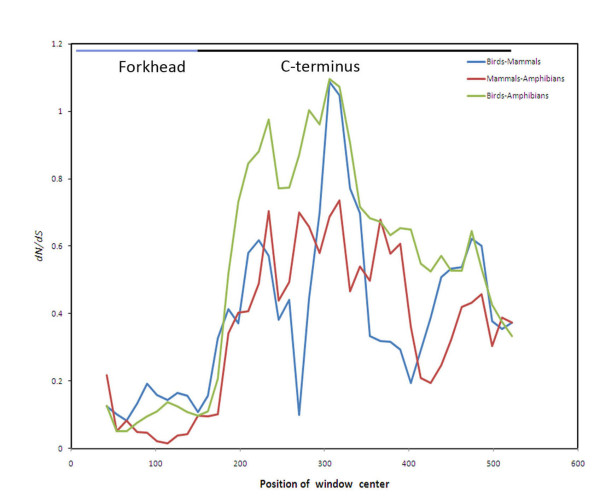
**Sliding window analysis of cumulative dN/dS across birds and amphibians, birds and mammals, mammals and amphibians**. The analysis is given across the coding regions of *FOXE1 *genes. In each case, the window was set at 60 bps and step 12 bps.

As an alternative analysis, we also estimated the average dN/dS profile in the sliding window analysis across FOXE1 sequences of three pairs of species groups - birds and mammals, birds and amphibians, and, mammals and amphibians (Figure 5). A low dN/dS ratio is estimated for the forkhead domain in all groups, which is indicative of a strong purifying selection. In contrast, the dN/dS profile shows a large variation in the C-terminal region of FOXE1. Nonsynonymous substitutions were predominantly concentrated within the C-terminus of FOXE1. In the two groups that included the birds, dN/dS is much more pronounced when compared to the amphibian and mammal groups.

Next, we applied the branch-site model to estimate the fraction of codons that are likely to have evolved under positive selection specifically in the five avian branches using the BEB procedure implemented in PAML. We used the five avian branches as the foreground and the other branches as background. The test compares two models: (1) no change in selection was observed in the foreground branches compared to the background branches, and (2) a certain proportion of sites went from being under negative or no selection in the background branches to being under positive selection in the foreground branch. The LRT test statistic (2Δ*l*) of the second model relative to the first model was 13.65 (*P *= 0.001, *df *= 2), indicating that a certain fraction of sites did undergo positive selection specifically in the avian lineages. Based on the Bayesian posterior probabilities (*BEB*) of site class, this analysis detected nineteen sites with *BEB *≥ 0.5. Of those, six sites were selected in the branch leading to *Galliformes*; however, the *BEB *values for these were less than 0.8. Two of the sites, 196Q and 203L, were detected to be adaptively evolving in bird lineages with probability > 0.95. There were seven additional sites with *BEB *≥ 0.8, which are 134R (0.927), 162A (0.858), 164R (0.898), 173P (0.923), 175P (0.884), 211P (0.80) and 249R (0.89). The amino acid coordinates are provided relative to the chicken FOXE1 protein. These results indicate a change in selective pressure on specific amino acids on the branch leading to birds.

**Figure 6:**
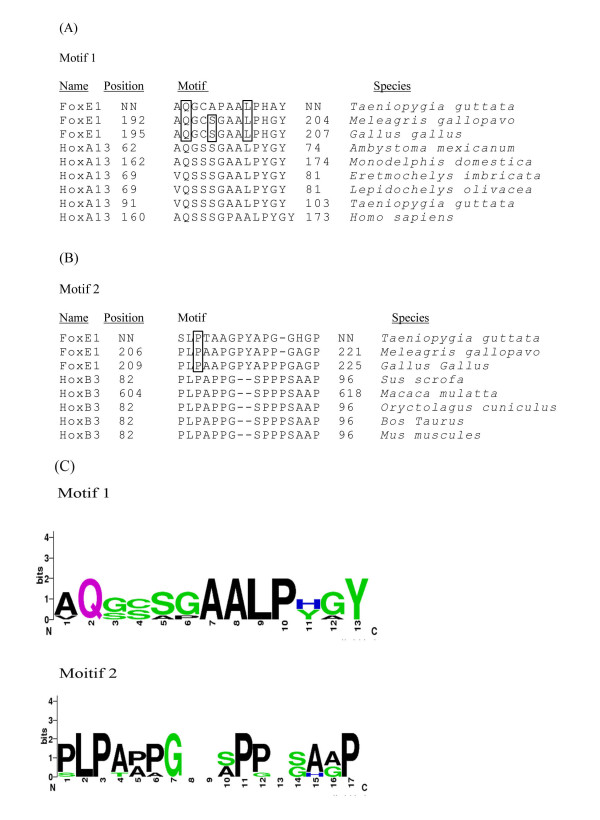
**Multiple sequence alignments of the avian FOXE1 and HOX protein sequences, which share similarity**. (A) The sequences of the avian FOXE1 proteins, which share similarity to the N-terminal sequence of the HOXA13 proteins. (B) The sequence of the avian FOXE1 showing the similarity to HOXB3 proteins. The positively selected sites are shown in box. NN- represents the sequence which has an undetermined 5'- end. (C) Consensus sequences of the identified motifs, shared between avian FOXE1 and HOX proteins were generated with the program Logo. The data for these logo sequences were generated based on six protein sequences. Gaps in the consensus sequence indicate alignment gaps. The logo sequences were generated with the WebLogo program [46].

A single positively selected site, 134R, was identified within a forkhead DNA-binding domain, which resulted in the non-synonymous substitution of Arg to Glu. The rest of the positively selected residues were identified in the C-termius, which is likely to be involved in protein-protein interactions. It is known that the *trans*-regulatory domains of transcriptional factors are comprised of short co-factor interaction motifs. By adapting a BLAST search for short sequences, we searched for short C-terminal segments of chicken FOXE1, consisting of 20 residues with the positively selected sites. We found that segment 195-207 aa of the chicken FOXE1 protein exhibited a high similarity to the N-terminal region of the homeobox-related transcriptional factor HOXA13 (Figure 6A). The hit between FOXE1 and HOXA13 had the highest score of ~30 bits. This sequence contains two adaptively evolving sites, 196Q and 203L, with the highest posterior probability, and 199S with *BEB *= 0.75. Based on the highest scores and number of hits, the match is likely to be associated with transcriptional function. Interestingly, the majority of HOXA13 proteins that exhibited the match were from tetrapods; catfish HOXA13 was detected but with a lower log-odds score. The region of similarity in HOXA13 proteins is located in the N-terminal domain outside of the homeodomain. Therefore, it may be potentially associated with activation or protein interaction function. Another short segment, 209-225 aa matched short proline enriched sequences in homeobox proteins HOXB3 and HOXA4 in the BLAST search with a score of 30 bits (Figure 6B). This sequence contains a positive selected site, 211P with a *BEB *= 0.80. This short sequence is also enriched with proline and alanine residues, which are commonly found in minimal repression domains [22]. It is possible that this sequence can exhibit repressive activity and an increase of the proline residues can enhance repressive activity. The consensus of both sequences is shown on Figure 6C. In HOXB3 this sequence is located in the N-terminus outside of the homeodomain. The search of hits to other segments of avian FOXE1 proteins with the selected sites did not retrieve any significant matches to other transcriptional factors. Thus, we were able to identify two potentially functional sequences in avian FOXE1 proteins containing four positively selected sites.

## Discussion

In this study we report on the identification of the *FOXE1 *gene of three bird species and the characterization of *FOXE1 *expression pattern during chicken embryogenesis. Both *FOXE1 *of chicken and zebra finch are distinctively localized on the sex-determining Z chromosome, in contrast to placental and marsupial *FOXE1 *genes which are localized on autosomes. *In situ *hybridization shows that the expression of the chicken *FOXE1 *gene is restricted to the developing thyroid and feathers. Its thyroid expression is initiated at the stage of placode formation when the thyroid cells evaginate from the pharyngeal floor and migrate, and is also maintained during the stage of thyroid maturation. The process of evagination is characterized by tissue remodeling, which includes modulation of cell adhesiveness and cell mobility. Based on the pattern of *FOXE1 *expression, we propose that the transcription factor FOXE1 may regulate evagination of thyroid primordia by regulating specific genes required for cell motility and adhesiveness. This observation is supported by previous loss- and gain-of-function studies in the mouse and cell culture, respectively. For example, in *FOXE1*-null mice the secondary palate remains opened [7], which indicates inability of the palate shelves to adhere in the mutant mice [25]. Forced expression of mammalian *FOXE1 *in cell culture resulted in significantly increased expression of an actin-binding protein, tropomyosin isoform 3, and lower expression of integrin beta-1 and collagen type XI alpha-1 [26]. Tropomyosin has been shown to be important for regulating the actin mechanics in the cell cytoskeleton, and can mediate changes in cell morphology, adhesion and migration [27,28]. Similarly, integrin beta-1 has been shown to mediate cell migration [29]. Moreover, it has been recently shown that human FOXE1 directly regulates the signaling molecule TGF-3β [30], which in turn, is involved in regulation of cellular adhesion and extracellular matrix [31]. Thus, *FOXE1 *may be involved in regulation of a set of genes and signaling pathways that are required for controlling cell adhesiveness and motility during migration and morphogenesis of thyroid cells. In the future, it will be important to determine whether chicken *FOXE1 *directly regulates a similar set of genes during the migration and morphogenesis of the thyroid gland. We also detected expression of FOXE1 in the growing feather, which suggests the acquisition of a novel expression domain by *FOXE1 *in the bird ancestry; since the feather is a bird specific integumentary appendage.

Two striking features are found in the sequence of avian FOXE1 proteins, which are the sequence divergence of the C-terminus and the loss of two functional domains: a C-terminal aromatic domain and the Eh1 motif as a consequence of two microdeletions. Both domains appear to be involved in mediating transcriptional repression. The aromatic domain of the mammalian FOXE1 protein can inhibit transcription in cell culture when fused to a heterologous DNA-binding domain, thus acting as a transferable repression domain [3]. Nothing is known about direct targets of this repression domain. Interestingly, mammalian FOXE1 represses transcriptional activation mediated by PAX8 in cell culture, which suggests that it may directly interact with transcription factor PAX8, possibly via this repression domain [3]. We noted that the avian genomes lack transcription factor PAX8 (personal communication), which is important for thyroid formation in mammals [32]. This is consistent with an extensive loss of genes in the chicken genome [33]. Thus, it would be interesting to determine whether the loss of the aromatic repressive domain was associated with the loss of the PAX8 locus in birds.

The Eh1 motif is a conserved amino acid sequence [18], known to mediate physical interaction of other Fox proteins with Groucho/TLE co-repressors. FOXG1, SLP2 (FOXG), FOXD3 and FOXH1 have been shown to interact physically with Groucho/TLE co-repressors via the Eh1 motif ([19,20,34] Yaklichkin and Kessler, unpublished data). Strikingly, the Eh1 motif is conserved in all FOXE1 of fish, amphibians, and non-placental mammals, but it was lost in those of birds. Interestingly, a loss of the Eh1 motif is also observed in FOXE1 of placental mammals as an outcome of a microdeletion (Yaklichkin and Kessler, unpublished data). The loss of the Eh1 motif in the avian FOXE1 protein is likely to lead to the loss of Groucho/TLE recruiting activity mediated by the Eh1 motif, and the loss of specific repressive activity dependent on the aromatic domain. Even though the functional implication of both domain losses in avian FOXE1 proteins is not clear, it is likely to affect transcriptional function. It is intriguing that the loss of two putative repressive domains is accompanied by a gain of an N-terminal polyalanine repeat. The avian FOXE1 domain losses may be associated with either functional divergence, loss of co-factor interacting proteins, or even reduction of expression domain. *In situ *hybridization in the chicken embryo shows that expression of *FOXE1 *is restricted to the developing thyroid and feathers, and no expression was observed in other internal embryonic tissues. *FOXE1 *orthologs have additional domain expressions other than in developing thyroid. For instance, frog foxe1 is expressed both in the developing thyroid and pituitary [9]. Foxe1 of zebrafish is expressed in pharyngeal skeleton, gills and thyroid [10]. It is certainly possible that the reduction of expression of *FOXE1 *in birds has resulted in the loss of these functional sequences. Similarly, a loss of the Eh1 motif in FOXE1 of placental mammals can possibly be associated with a novel functional requirement.

To investigate the role of selection in the evolution of *FOXE1 *coding regions in the avian lineages, we used various models of codon evolution dN/dS (ω). Overall, dN/dS (ω) of *FOXE1 *was estimated to be less than 1, which suggested that *FOXE1 *were evolving under purifying selection. Significant increase of the dN/dS ratio was estimated between the branches of avian *FOXE1 *and those of mammals and amphibians, which is indicative of a change in the selection and of the acceleration of evolution of avian 3'*FOXE1*. The increase of the dN/dS ratio can be a result of either a relaxation of purifying selection or positive selection in specific sites of the C-terminal domain of FOXE1 in the avian lineage. In paralogous regulatory genes, the relaxation of purifying selection was proposed to be a result of paralogous proteins binding to a subset of interacting proteins relative to the ancestral gene copy [35]. By this analogy, relaxation of purifying selection in avian *FOXE1 *could be a result of loss of ancestral protein interactions and possibly formation of interaction with novel binding proteins. Overall, the C-terminal domain is subjected to fewer functional constraints when compared to the DNA-binding forkhead domain. An increased evolutionary rate of C-terminal regions can be attributed to the capacity of *trans*-regulatory domains to interact with co-factors and the transcriptional machinery via short interaction motifs. In turn, interaction peptide motifs can evolve quickly due to short size and low affinity of interaction with co-factors [36].

Our branch-site model identified eighteen C-terminus residues under positive selection in the avian lineage, and two residues, 196Q and 203L, had the highest posterior probability, suggestive of adaptive evolution. Only a single adaptively evolving residue, 134R, was identified in the forkhead DNA-binding domain, which resulted in a non-synonymous substitution in the avian lineage, whereas all other residues lie in the C-terminus. Interestingly, the 196Q and 203L residues are located in a segment (195-207 aa) of avian FOXE1 proteins. This segment shows a strong homology to N-terminal short sequences of the homeobox HOXA13 proteins. The N-terminal portion of the HOXA13 protein contains a *trans*-regulatory domain, which is likely involved in regulation of transcription. Mouse HOXA13 has been shown to function as a negative regulator [37]. Moreover, HOXA13 can inhibit Smad-mediated activation of transcription by binding directly to Smad co-factors via the N-terminus [38]. However, refined mapping of Smad-interacting sequences have not been conducted. It is likely that the region (195-207 aa) is involved in regulation of transcription based on high homology to the N-terminal segment of homeodomain-containing HOXA13 protein, and adaptively evolving residues may have contributed to avian specific FOXE1 function.

A residue 211P under positive selection was found in the FOXE1 segment (214-225 aa) enriched with proline and alanine residues. This segment shares a high similarity with N-terminal sequences of HOXB3 and HOXA4 proteins of mammals, which are also enriched with proline and alanine residues. Interestingly, the mouse HOXB3 protein can function as a transcriptional repressor [39], and is expressed in the thyroid primordia and regulates its migration [40]. Minimal repression domains of metazoan transcription factors are known to be often enriched with proline and alanine residues [22]. It is thus predicated that this region may be involved in repression of transcription. It is possible that the avian-specific gain of proline residues has contributed to enhancement of repressive characteristics or the formation of novel avian-specific motifs. Additionally, we cannot exclude the contribution of the N-terminus to transcriptional activity of avian FOXE1, which has gained polyalanine repeats. Thus, relaxed selection in the avian lineage may be the predominant contributor to the accelerated evolution of avian FOXE1 and significant sequence divergence of the C-terminus, whereas a limited positive selection could lead to the formation of novel avian specific transcriptional motifs.

Evolution of gene expression, and thus, the evolution of transcription factors, is likely to play a major role in morphological evolution. Because of the pleiotropic effects of changes in transcription factor sequence, some have argued that changes in gene regulatory networks are predominantly mediated via changes in DNA *cis*-elements [41]. However, negative pleiotropic effects can be limited by tissue-restricted expression of transcription factors and changes in the transcription factor sequences affecting their interaction with other tissue-specific co-factors [42]. This seems to be the case for FOXE1 evolution in birds. However, directed experiments will be needed to further clarify the functional underpinnings of the evolutionary divergence of avian FOXE1.

## Conclusions

Identification of functional *FOXE1 *orthologs and their codon analysis can provide important insight into their contribution to vertebrate evolution, and offer a foundation for the study of their function across vertebrates. Comparative biochemical studies will be necessary to determine transcriptional function and the effect of the loss of two functional domains in comparison to the other FOXE1 proteins. Building on ongoing functional and structural studies should yield a comprehensive understanding of the evolution of *FOXE1 *in vertebrates.

## Methods

### DNA and protein sequences

DNA sequences of *FOXE1 *were obtained from the NCBI database http://ncbi.nlm.nih.gov and the Ensembl database (http://ensembl.org, v47). The *FOXE1 *sequences of chicken, zebra finch and turkey identified in this study were deposited under the accession numbers BK008024, BK008025 and AEE88205, correspondently at the NCBI database. The accession numbers of *FOXE1 *sequences obtained from the NCBI are following: *Homo sapiens *(NP_004464), *Mus musculus *(NP_899121.1), *Xenopus laevis *(AAS82575.1), *Xenopus tropicalis *(XP_002936729.1), *Macropus eugenii *(ADN52078.1), *Monodelphis domestica *(XP_001372714.1), and *FOXE1 *of *Sus scrofa *(ENSSSCT00000005909) was obtained from the Ensembl database v47. The sequence of chicken *FOXE1 *was obtained from a Z chromosome BAC sequence (AC192757.2) in NCBI. The sequence of zebra finch *FOXE1 *was obtained from the chromosomal Z region - 31551529-31552116 bp (genome version WUGSC 3.2.4/taeGut1), from UCSC Genome Browser http://genome.ucsc.edu.

### Sequence and phylogenetic analysis

Analysis of amino acid composition of deduced FOXE1 protein sequences was performed using the SAPS program (isrec.isb-sib.ch/software/SAPS_form.html; [21]). A search of *FOXE1 *in the NCBI chicken genome database was performed using the BLAST server http://blast.ncbi.nlm.nih.gov. Identification of the Eh1 motif in *FOXE1 *sequences was performed in accordance with the previously described sequence analysis [18]. Multiple sequence alignments were constructed using T-COFFEE, version 7.7.1. (tcoffee.vital-it.ch/cgi-bin/Tcoffee/tcoffee_cgi/index.cgi; [43]). Indels (small insertion or deletion mutations/sequencing errors) in the aligned sequences were removed using the alignment editor BioEdit 7.0.4.1. http://www.mbio.ncsu.edu/bioedit/page2.html. Syntenic alignment was generated by comparing the surrounding genomic region in ensembl and metazome http://www.metazome.net. A phylogenetic tree of *FOXE1 *genes was constructed by using the software Phylip 3.69 [44]. The phylogenetic tree was converted into a cladogram using MEGA 4 http://www.megasoftware.net/.

### dN/dS analysis

The dN/dS (ω) analysis was performed using the program *Codeml *in the PAML package 3.13 [23] to assess whether FOXE1 evolved under a differential selection in the avian lineage, relative to the rest of the phylogeny. We specifically performed the analysis on the C-terminus of the avian FOXE1 proteins. Positive selection on specific bird lineages were tested using branch models. A model in which ω was fixed across the tree (one-ratio) was compared with models in which ω was allowed to differ in a subset of branches (two- and three-ratio models), and the significance of the difference was assessed using the likelihood ratio tests (LRTs).

To identify the sites under positive selection along the avian *FOXE1 *genes, we used the branch-site model A. We used the avian branch set as the foreground branches and all other branches as the background. We then tested whether a model which allows a subset of background sites under neutral or purifying selection, to evolve under neutral or positive selection in the foreground. Model significance was tested using the LRTs termed Test 1. In Test 1 branch-site model A is compared with two degrees of freedom to a site model (M1a, "Nearly Neutral") with two site classes: 0 < ω0 < 1 and ω2 = 1. In addition, PAML also computes for each site the posterior probability of belonging to the class that undergoes an increase in dN/dS.

To visualize variation in ω along *FOXE1 *genes, a slide window analysis was conducted using the software SWAAP 1.0.3. http://asiago.stanford.edu/SWAAP/SwaapPage.htm. A window size was set to 60 bps and the step size to 12 bps. Values of ω were estimated in accordance with the Nei and Gojobori method.

### Polymerase Chain Reaction (PCR) Amplification and Sequencing of genomic DNA

Turkey blood was kindly donated by Bolton Turkey Farm, Silverdale, PA. Genomic DNA was isolated from the blood using the QIAamp DNA Blood Midi Kit (cat. number: 51183). For amplification of turkey *FOXE1*, pairs of primers were designed to regions with 100% identity shared between chicken and zebra finch *FOXE1 *genes, and upstream and downstream regions of chicken *FOXE1*. Primer sequences are available by request. PCR fragments covering the single exon *FOXE1 *were amplified using GC-RICH PCR System (cat. number: 12 140 306 001), Roche Applied Science. PCR mixture was made in accordance with the protocol of the maker. The following conditions in PCR reactions were used: Initial denaturation, 3 min. at 95°C; 1 step - denaturation - 20 sec. at 95°C; 2 step - primer annealing at 60°C, 30 sec.; 3 step - elongation - 1 min. at 68°C, and the final elongation for 7 min. at 68°C. 35 cycles were performed for the amplification of turkey *FOXE1*. PCR fragments were isolated from 1-1.5% agarose gel using the QIAquick gel Purification Kit (cat. number: 28704) and sequenced by a cycle sequencing reaction by Sanger's dideoxy Terminator Method on a PCR Machine. The sequence of the entire turkey *FOXE1 *gene and flanking regions was assembled by using the program ApE, a plasmid editor v.1.17.

### Molecular cloning and in situ hybridization with FoxE1 GC-rich probe

To clone a chicken *FOXE1 cDNA*, mRNA was isolated from day 5 chicken embryos using the Qiagen RNeasy Mini Kit (cat. number: 74104). cDNA was synthesized using 1 μg of RNA per a reverse Transcription (RT) reaction with the Tetro cDNA Synthesis Kit (cat. number: Bio-65042). PCR was used for template generation with the GC-rich PCR System Kit (cat. number: 12 140 306 001), Roche Applied Science; 34 cycles of annealing at 50°C were performed. PCR products were isolated and purified with the QIAquick Purification Kit (cat. number: 28106). All protocols were performed in accordance with kit instructions The cDNA template for generating the chicken *FOXE1 *antisense RNA *in situ *hybridization probe was produced by RT-PCR using the following primers: forward primer, 5'TTATAAAAGCTTGCGGCCGCAGAATAT**CGGCAAGGGCAACTACTGGAC**3'; reverse primer, 5'GCTCTAGAA*ATTAACCCTCACTAAAGG***gcggggacgaacctGTCG**3'. Chicken *FOXE1 *sequences are bolded, the T3 RNA polymerase binding site is italicized, remaining sequence contains restriction sites (PsiI, HindIII, NotI, XbaI). The PCR generated cDNA template was sequenced to confirm identity. A standard 504 bps RNA probe of *FOXE1 *was produced using T3 polymerase and *in situ *hybridizations were performed in according to the GEISHA mRNA Detection Protocol http://geisha.arizona.edu/geisha/protocols.jsp[45].

## Authors' contributions

SY and SH conceived this study. SY, SH and DD designed the experiments. DD and MP carried PCR of chicken *FOXE1 *probe and *in situ *hybridization on chicken embryos. SY and SH obtained and analyzed computational data, and DD and MP analyzed *in situ *hybridization data. SY isolated turkey genomic DNA and sequenced turkey *FOXE1 *gene. SY, SH and DD wrote the manuscript. All authors read and approved the final manuscript.

## Supplementary Material

Additional file 1 **Supplementary Tables S1 and S2, and Supplementary Figures S1-S4**. Supplementary Table S1. Amino Acid Composition of the chicken FoxE1 protein. Supplementary Table S2. Separated and tandem repeats in the chicken FoxE1 protein. Supplementary Figure S1: Nucleotide and deduced amino acid sequences of the chicken *FOXE1 *gene. Supplementary Figure S2: The GC-content along the chicken *FOXE1 *gene. Supplementary Figure S3: Nucleotide and deduced amino acid sequences of the zebra finch *FOXE1 *gene. Supplementary Figure S4: Sequence alignment of the FOXE1 proteins of chicken and zebra finch. Click here for file

Additional file 2 **Supplementary Figure S5**. Supplementary Figure S5: Sequence alignment of FOXE1 proteins. Click here for file
