# Supplementary material for: Accelerated evolution of 3'avian FOXE1 genes, and thyroid and feather specific expression of chicken FoxE1
Source: BMC Evol Biol. 2011 Oct 15;11:302. doi: 10.1186/1471-2148-11-302 (PMC3207924; doi:10.1186/1471-2148-11-302)
Supplement: Additional file 1 — Supplementary Tables S1 and S2, and Supplementary Figures S1-S4. Supplementary Table S1. Amino Acid Composition of the chicken FoxE1 protein. Supplementary Table S2. Separated and tandem repeats in the chicken FoxE1 protein. Supplementary Figure S1: Nucleotide and deduced amino acid sequences of the chicken FOXE1 gene. Supplementary Figure S2: The GC-content along the chicken FOXE1 gene. Supplementary Figure S3: Nucleotide and deduced amino acid sequences of the zebra finch FOXE1 gene. Supplementary Figure S4: Sequence alignment of the FOXE1 proteins of chicken and zebra finch. [file 1471-2148-11-302-S1.DOC]

**Supplementary Tables**

**Supplementary Table 1.** Amino Acid Composition of the chicken FOXE1 protein. Amino acid frequencies were elevated for the chicken FoxE1 sequence of 290 aa (Brendel et al. 1992). The 1% quantile is indicated by the label --; low usage in the 5% quantile is indicated by the label `-'; high usage above the 95% quantile point is indicated by the label `+', high usage above the 99% quantile point is indicated by the label `++'. Frequencies of quantiles were estimated with respect to the set of chicken proteins.

| **Amino Acid** | **Count (Frequency)** | **Amino Acid** | **Count (Frequency)** |
| --- | --- | --- | --- |
| A++ | 51(17.6%) | P+ | 52(17.9%) |
| G | 30(10.3%) | V- | 5(1.7%) |
| M | 3(1.0%) | E | 10(3.4%) |
| S | 14(4.8%) | K | 8(2.8%) |
| C | 5(1.7%) | Q- | 5(1.7%) |
| H | 4(1.4%) | W | 2(0.7%) |
| N- | 4(1.4%) | F | 13(4.5%) |
| T- | 6(2.1%) | L | 22(7.6%) |
| D | 8(2.8%) | R++ | 30(10.3%) |
| I- | 6(2.1%) | Y | 12(4.1%) |

**Supplementary Table 2.** Separated and tandem repeats in the chicken FOXE1 protein.

| **Amino acid position** | **Repetitive element** |
| --- | --- |
| 165-168 | PAAP |
| 182-185 | PAAP |
| 211-214 | PAAP |
| 180-183 | PAPA |
| 240-243 | PAPA |
| 215-220 | GP_YAPP |
| 224-230 | GPLYAPP |
| 216-219 | PYAP |
| 253-256 | PYAP |

**Supplementary Figures**

**Supplementary Figure 1**. Nucleotide and deduced amino acid sequences of the chicken *FOXE1* gene. The start and stop codons are italicized. The forkhead domain and polyalanine repeat are underlined, respectively.


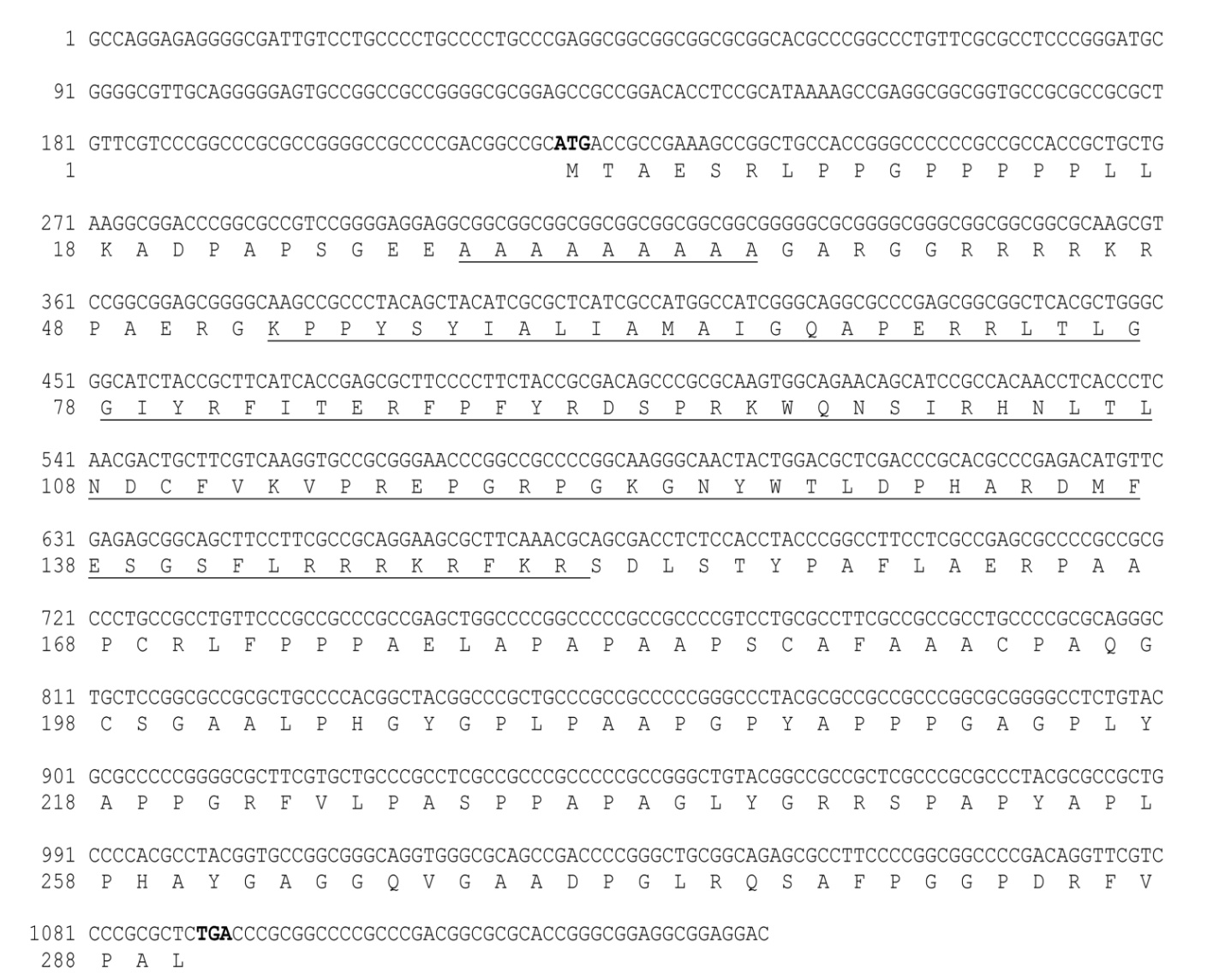


**Supplementary Figure 2**. The GC-content along the chicken *FOXE1* gene.


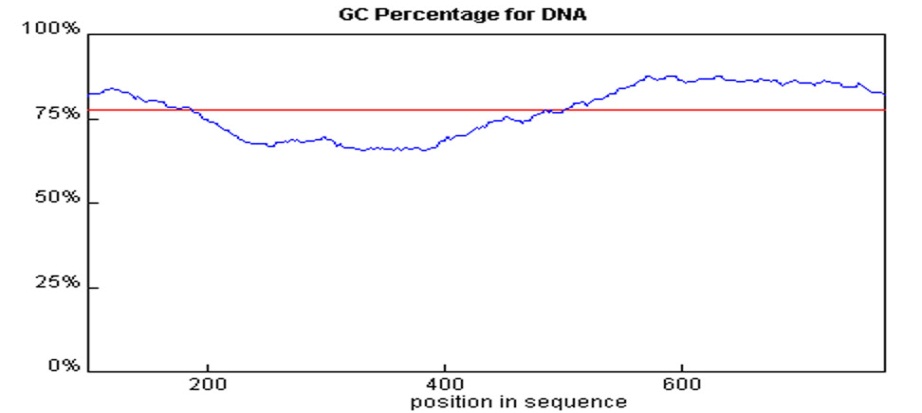


**Supplementary Figure 3.** Incomplete nucleotide and deduced amino acid sequences of the zebra finch *FOXE1* gene. The forkhead domain is underlined.


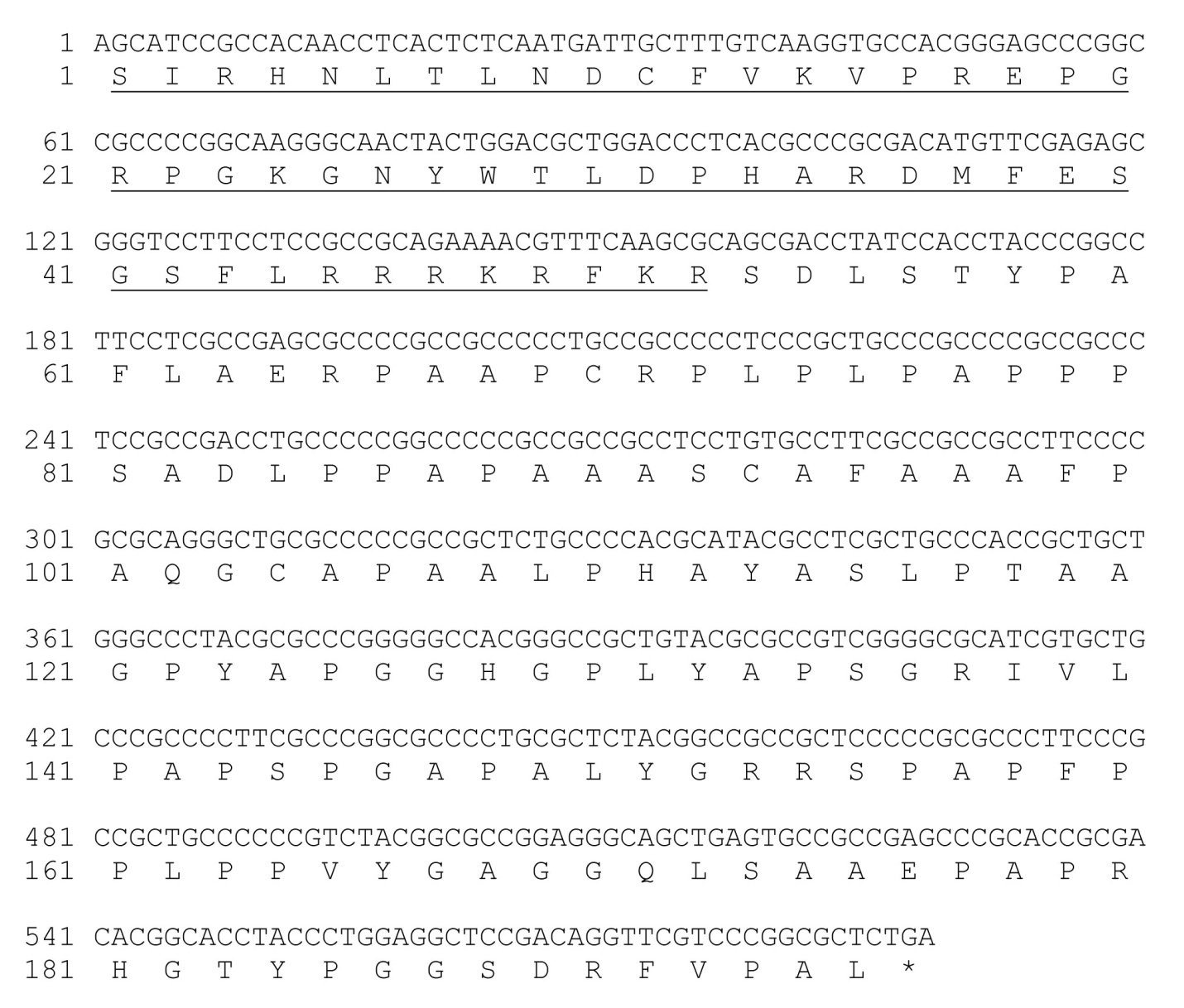


**Supplementary Figure 4.** Sequence alignment of the FOXE1 proteins of chicken and zebra finch. Abbreviation: FoxE1_chk: FoxE1 of the chicken and FoxE1_zeb of zebra finch.
