# Supplementary material for: Accelerated evolution of 3'avian FOXE1 genes, and thyroid and feather specific expression of chicken FoxE1
Source: BMC Evol Biol. 2011 Oct 15;11:302. doi: 10.1186/1471-2148-11-302 (PMC3207924; doi:10.1186/1471-2148-11-302)
Supplement: Additional file 2 — Supplementary Figure S5. Supplementary Figure S5: Sequence alignment of FOXE1 proteins. [file 1471-2148-11-302-S2.DOC]

**Supplementary Figure 5.** Sequence alignment of the FOXE1 proteins. The avian polyalanine repeat is underlined.

FoxE1_chic MTAESRLPPG P--------- --PPPP---- ---------- LLKADPAPSG

FoxE1_turk MTAESRLPPG P--------- -PPPPP---- ---------- PLKADPAPPG

FoxE1_zebr ---------- ---------- ---------- ---------- ----------

FoxE1_Homo MTAESGPPPP Q--------- -P---E---- ---------- VLATVKEERG

FoxE1_rat MTAESAPPPP P--------- ---QPE---- ---------- ALAAVKEERG

FoxE1_pig MTAESGPPPP P--------- -PQQSE---- ---------- ALAAVKQERG

FoxE1_Mus MTAESAPPPP P--------- ---QPE---- ---------- TLAAVKEERG

FoxE1_oppo MTEERRHSPQ RDCTEDQDLP HPPTPVAEAA GR--TLTLMP VVKVEKEPPA

FoxE1_kang MTEERRRSPQ KDRPEDRGLQ HPPTPVAEAA GR--SLPLMP VVKVEKEPAA

FoxE1_trop MTAESQQSPT R--------- ---ATAAGAG LQQTSGFTMP VVKVEKDPAP

FoxE1_Xeno MTAESQQSPT R--------- ---ATAAGAG LQQTSGFTMP VVKVEKDPAP

FoxE1_fugu M--------- ---------- ---------- ---------P VVKVEKESSA

FoxE1_tetr M--------- ---------- ---------- ---------P VVKLEKDSLA

FOXE1_MEDA M--------- ---------- ---------- ---------P VVKVEKDSQS

FOXE1_STIC MT-------- ---------- ---------- --------MP VVKVEKEPQA

FoxE1_Dani M--------- ---------- ---------- ---------P VVKVESDSPS

EE**A-A**----- **AAAAAAAG-A** RGGRRRRKRP AERGKPPYSY IALIAMAIGQ

EE**A**------- ---**AAAAG-A** RGGRRRRKRP AERGKPPYSY IALIAMAIGQ

---------- ---------- ---------- ---------- ----------

ETA-A---GA GVPGEATG-R GAGGRRRKRP LQRGKPPYSY IALIAMAIAH

EAA-A---GA GVPAEVAG-R GAGGRRRKRP LQRGKPPYSY IALIAMAIAH

EA------GA GVPAEAAG-R GAGGRRRKRP LQRGKPPYSY IALIAMAIAH

EAAAA---GA GVPAEAAG-R GAGGRRRKRP LQRGKPPYSY IALIAMAIAH

CEPSGGLSEL GEPVTKASG- GGGGRRRKRP LQRGKPPYSY IALIAMAIAH

CEPSGGLSEL GEPAAKAGSG GGGGRRRKRP LQRGKPPYSY IALIAMAIAH

EAS-M----- SNGGSEVDD- THKGRRRKRP LQKGKPPYSY IALIAMSIAN

EAS-M----- SNGGSEVDD- THKGRRRKRP LQKGKPPYSY IALIAMSIAN

ENP-P---PA SNLPQQTEE- QSRGRRRKRP LQQGKPPYSY IALISMAIAN

ENP-P---PA SNLTQQTEE- QPRGRRRKRP LQQGKPPYSY IALISMAIAN

DAV-L---PT SDPPPQTEE- QPRGRRRKRP LQRGKPPYSY IALISMAIAN

EHK-L---PA SNPSVQSEE- QPRGRRRKRP LQRGKPPYSY IALISMAIAN

ETT-L---PV N--DSQRAE- PQRGRRRKRP LQRGKPPYSY IALISMAIAN

APERRLTLGG IYRFITERFP FYRDSPRKWQ NSIRHNLTLN DCFVKVPREP

APERRLTLGG IYRFITERFP FYRDSPRKWQ NSIRHNLTLN DCFVKVPREP

---------- ---------- ---------- -SIRHNLTLN DCFVKVPREP

APERRLTLGG IYKFITERFP FYRDNPKKWQ NSIRHNLTLN DCFLKIPREA

APERRLTLGG IYKFITERFP FYRDNPKKWQ NSIRHNLTLN DCFLKIPREA

APERRLTLGG IYKFITERFP FYRDNPKKWQ NSIRHNLTLN DCFLKIPREA

APERRLTLGG IYKFITERFP FYRDNPKKWQ NSIRHNLTLN DCFLKIPREA

APDRKLTLGG IYKFITERFP FYRDNPKKWQ NSIRHNLTLN DCFIKIPREP

APDRKLTLGG IYKFITERFP FYRDNPKKWQ NSIRHNLTLN DCFIKIPREP

SADRKLTLGG IYKFITERFP FYRDNSKKWQ NSIRHNLTLN DCFIKIPREP

SADRKLTLGG IYKFITERFP FYRDNSKKWQ NSIRHNLTLN DCFIKIPREP

SPDRKLTLGG IYKFITERFP FYRDNSKKWQ NSIRHNLTLN DCFIKIPREP

SPDRKLTLGG IYKFITERFP FYRDNSKKWQ NSIRHNLTLN DCFIKIPREP

SPDRKLTLGG IYKFITERFP FYRDNSKKWQ NSIRHNLTLN DCFIKIPREP

SPDRKLTLGG IYKFITERFP FYRDNSKKWQ NSIRHNLTLN DCFIKIAREP

SPDRKLTLGG IYKFITERFP FYRDNSKKWQ NSIRHNLTLN DCFIKIPREP

GRPGKGNYWT LDPHARDMFE SGSFLRRRKR FKRSDLSTYP AFLAERPAA-

GRPGKGNYWT LDPHARDMFE SGSFLRRRKR FKRSDLSTYP AFLAERPAA-

GRPGKGNYWT LDPHARDMFE SGSFLRRRKR FKRSDLSTYP AFLAERPAA-

GRPGKGNYWA LDPNAEDMFE SGSFLRRRKR FKRSDLSTYP AYMHDAAAAA

GRPGKGNYWA LDPNAEDMFE SGSFLRRRKR FKRSDLSTYP AYMHDAAAAA

GRPGKGNYWA LDPNAEDMFE SGSFLRRRKR FKRSDLSTYP AYMHDAAAAA

GRPGKGNYWA LDPNAEDMFE SGSFLRRRKR FKRSDLSTYP AYMHDAAAAA

GRPGKGNYWA LDPNAEDMFE SGSFLRRRKR FKRTDLSTYP AYMHDAAAAA

GRPGKGNYWA LDPNAEDMFE SGSFLRRRKR FKRTDLSTYP AYMHDAAAAA

GRPGKGNYWA LDPNAEDMFD SGSFLRRRKR FKRTDLTTYP AYIHDTSMFS

GRPGKGNYWA LDPNAEDMFD SGSFLRRRKR FKRTDLTTYP AYIHDTSMFS

GRPGKGNYWA LDPNAEDMFE SGSFLRRRKR FKRCDFSTYT SYMHEAPVFS

GRPGKGNYWA LDPNAEDMFE SGSFLRRRKR FKRSDFSTYN SYVHETPVFP

GRPGKGNYWA LDPNAEDMFE SGSFLRRRKR FKRCDLSTYA SYVHDTPVFS

GRPGKGNYWA LDPNAEDMFE SGSFLRRRKR FKRCDLSTYT SYVHETPVFS

GRPGKGNYWA LDPNAEDMFE SGSFLRRRKR FKRSDFTTYS SYVHESPVFS

---------- ---------- ---------- ---------- ----------

---------- ---------- ---------- ---------- ----------

---------- ---------- ---------- ---------- ----------

AAAAAAAAAA AIFPGAVPAA RP-PYPGAVY AGYA----PP ----SLAAPP

AAA-----AA AIFPGAVPAA RP-AYPGAVY AGYA----PP -----LAAPP

AAAA-AAAAA AIFPGAVPAA RP-PYPGAVY ASYA----PP ----SLAAPP

AAA-----AA AIFPGAVPAA RP-AYPGAVY AGYA----PP -----LAAPP

AAAA----AA GMFPSSVPVA RP-PYPGSVY PNVAAAMSPA GYGQTLGPHS

AAAA----AA GMFPSSVPVA RP-PYPGSVY PNVAAAMNPA GYGQTLGPHS

P--------- ------LQVA RA-TYPNTVY PNMTM--SP- NYSQQIAPHS

P--------- ------LQVA RA-TYPNTVY PNMTM--SP- NYSQQIAPHS

P--------- ------VQIA RSAAYANSVY PNMAV--GP- AYGQQL---P

P--------- ------VQVT RSAVYTNSVY PNMAV--GP- AYGQQL---P

P--------- ------VQIP RSAAYSNPVY PNMTV--SP- TYGQQL---P

P--------- ------LQIA RSTAYTNSVY NNMAV--SP- AYGQQL---T

P--------- ------VQIA RS-AYANSVY SNMAV--SP- PYAQQL---P

---------- -----PCRLF P--------- ---------- ---------P

---------- -----PCRLF P--------- ---------- ---------Q

---------- -----PCRPL PL-------- ---------- -----PAPPP

PVYYPAASP- ----GPCRVF GLV------- ---------- -----PERPL

PVYYPAASP- ----GPCRVF GLV------- ---------- -----PERPL

PVYYPAASP- ----GPCRVF GLV------- ---------- -----PERPL

PVYYPAASP- ----GPCRVF GLV------- ---------- -----PERPL

SVYYPASSP- ----GQCRVF SINSLVHGPG D--L-LQQQP APPPALGRPL

SVYYPASSP- ----GQCRVF SINSLVHGPG D--L-LQQQP APPPALGRPL

SVYYPSSSP- AFSSAQPRVF SINTLIGHSG ---------S EHAQPPNRSI

SVYYPSSSP- AFSSAQPRVF SINTLIGHSG ---------S EHAQPPNRSI

SAYYPSSSPS GFSPGQARMF SINNIIGNPA AAGMMGGQGP EAAQQSSRSF

SAYYPSSSPS GFSPGQARMF SINNLIGHPA AAGMLGGQGP EVVQQSNRSF

SAYYPSPSPP GFAHSQTRMF SINNIIGHPS AASMLANQGV DGMQQPSRTF

SAYYPSSSPP GFGPGQTRMF SINNLIGHQT PGSMLGGQVP EAMQQPGRSF

SAYYQSSSP- NFTAGQSRVF RINSLIGSPS RM---GQNAE MIPQQSCRSF

PAELAPAPA- -APSCAFAAA CPAQ------ --GCSGA--- ----------

PAELAPAPA- -APSCAFAAA CPAQ------ --GCSGA--- ----------

SADLPPAPA- -AASCAFAAA FPAQ------ --GCAPA--- ----------

SPELGPAPSG PGGSCAFASA GAPATTTGYQ PAGCTGA--- ----------

SPDLGPAPSA AGGSCAFAAA AGAPGTGSFQ PAVCTGA--- ----------

SPELGPSPSG PVGSCAFASA GASAATTGYQ PTGCAGA--- ----------

SPDLGPAPSA AGGSCAFAAA AGAAGTGSFQ PAVCTGA--- ----------

SPDLSQAPSV AAGSCSFGAS AAAAAA-SYN NPACSGGSGG GGGAGPAGVL

SPDLSQAPSV AAGSCSFGAS AAAAAA-SYS NPACSGGSGG GGGAGAAGVL

SPEVNSTS-- -SSSCNYGGS A-------YS SQAGSGTML- ----------

SPEVNSTS-- -SSSCNYGGS A-------YS SQAGSGTML- ----------

SPEGHPNE-- -SNPCNLGGP A-------FQ GQTCGAG-S- ----------

SPEGHPNE-- -SNPCNLGGP A-------FQ GQTCSAV-S- ----------

SPEGLMNG-- -STPCGLGAP S-------YQ GQSCGGTVA- ----------

SPEGPPNG-- -SGPCGLAAP A-------FH SQPCGGPVS- ----------

SPE------- -SGSCSLGGP G-------FQ HQSCNGETV- ------LSCY

-A--LPHGYG PLPA-A---- -----PGPYA PPPGAGPLY- APPGRFVLPA

-A--LPHGYG PLPA-A---- -----PGPYA -PPGAGPLY- APPGRFVLPA

-A--LPHAYA SLPT-A---- -----AGPYA -PGGHGPLY- APSGRIVLPA

-RPANPSAYA AAYAGP---- -----DGAYP -QGAGSAIF- AAAGRLAGPA

-RPVNPAAYA AAYAGP---- -----DGAYP -QGASSALFA AAAGRLAGPT

-RPANPSAYA AAYAGP---- -----DGAYP -QGASSALF- AATGRLAGPS

-RPVNPAAYA AAYAGP---- -----DGAYP -QGASSALFA AAAGRLAGPA

PRPSNPMAY- -TYPVPNGHL --PMNHGSYP -QGNSSQLF- GASGRLAMST

PRSSNPMAY- -TYPVPNGHL --PMNHGSYP -QGNSSQLF- GASGRLAMST

PRSTNPVPY- -SYSVPNSHL --QMNQSTYT -HSN-AQLF- GSASRLPMPT

PRSTNPVPY- -SYSVPNSHL --QMNQSTYT -HSN-AQLF- GSASRLPMPT

SRSAAHPSF- -TYSGQNVHQ HHHTHQSSYG -QGH-TQGY- AVAGRLHSSS

SRAATHPTF- -TYSGQNVHQ HHHTHQSSYG -QGH-TQGY- AVAGRLHSSS

PRSTAHPGF- -NYSAPSSHS H--HHQSSYG -QGS-TQSY- PPAGRIHASA

SRSSAHPGF- -NYSGPNSHP HQHPHQGSYG -QGQ-TQGY- AATGRLHPSA

SSSSNNMAF- -AYSGPG-H- --GQTQVSYP -QAS-TQHY- GPAGRMAISS

SPPA------ ---P-AGLYG RRSPAPYAP- LPHAYGA-GG QVGAADPG--

SPPA------ ---P-AGLYG RRSPAPYAP- LPHAYGA-GG QVGAADPG--

PSPG------ ---A-PALYG RRSPAPFPP- LPPVYGA-GG QLSAAEPA--

SPPAGGSSGG VETT-VDFYG RTSPGQFGA- LGACYNP-GG QLGGASAGAY

SPTAGGGSGG VEAT-VDFYG RTSPGQFGAA LGPCYNP-SG QLGAGGGGAY

SPPAGGSSGG VETA-VDFYG RTSPGQFGA- LAPCYNP-AG QLGTGSGGTY

SPPAGGGSGG VEAT-VDFYG RTSPGQFGAA LGPCYNP-GG QLGAGGGGAY

SPPGA----- -NDP-VDFYG RMSPGQISS- LAHSYNP-GG QLGAGPN-AY

SPPGG----- -NDP-VDFYG RMSPGQISS- LAHSYNP-GG QLGAGPS-AY

SPPMN----- -SDT-VDFYG RMSPGQYTS- LAT-YNS-NG QLGGTNAY--

SPPMN----- -SDT-VDFYG RMSPGQYTS- LAT-YNS-NG QLGGTNAY--

H--GP----- -TDS-VDHYG RVSPVQLGS- FSQ-YTAGGG PIASTGGY--

H--GL----- -TDS-VDHYG RVSPVQLGS- FSQ-YSTGGG PITSAGGY--

H--GP----- -AET-MDHYG RVSPVQLGS- FSH-YNS-PG PITNTGGY--

H-HGS----- -LEA-MDHYG RVSPVQLGS- FSQ-YNSAAG PIANTGGY--

LSPIA----- -GDAVGDPYG RTSPAQLGS- FVQ-YNN-SG AVGSSGAY--

-LRQS-AFPG GPDRFVPA-L

-LRQS-AFPG GPDRFVPA-L

-PRHG-TYPG GSDRFVPA-L

HARHAAAYPG GIDRFVSA-M

HSRHATAYPG AVDRFVSA-M

HARHAAAYPS GVDRFVSA-M

HSRHATAYPG AVDRFVSA-M

HVRHS-AYSS NVERFVSA-M

HVRHS-AYSS NVERFVSGHV

-LRHA-TYSG NMERFVPA-V

-LRHA-TYSG NMERFVPA-V

-LRHP-TYPG NMDRFVSA-I

-LRHP-TYPG NMDRFVSA-I

-LRHP-TYTG NIDRFVSA-I

-LRHP-TYPG NMDRFVSA-I

-IRHP-AYSG NMDRFVSA-T
